# Diversity and Distribution of Intertidal *Cystoseira sensu lato* Species Across Protection Zones in a Mediterranean Marine Protected Area

**DOI:** 10.3390/plants13243562

**Published:** 2024-12-20

**Authors:** Francesco Paolo Mancuso, Gianluca Sarà, Anna Maria Mannino

**Affiliations:** 1 Department of Earth and Marine Sciences (DiSTeM), University of Palermo, Viale delle Scienze Ed. 16, 90128 Palermo, Italy; 2 NBFC—National Biodiversity Future Center, 90133 Palermo, Italy; 3 Department of Biological, Chemical and Pharmaceutical Sciences and Technologies, University of Palermo, 90123 Palermo, Italy

**Keywords:** biodiversity, macroalgal distribution, Marine Protected Areas, Mediterranean Sea, *Cystoseira sensu lato*, *Cystoseira*, *Ericaria*, *Gongolaria*

## Abstract

This study investigates the diversity and distribution of intertidal *Cystoseira sensu lato* (*s.l.*) species across different protection zones within the “Capo Gallo-Isola delle Femmine” Marine Protected Area (MPA) in the central Mediterranean Sea. Five *Cystoseira s.l.* species (*Cystoseira compressa, C. foeniculacea, Ericaria amentacea, E. brachycarpa* and *E. crinita*) were observed on the intertidal rocky shores, with varied abundances across the MPA’s protection zones. *Ericaria amentacea* was the only species found in all zones, with a much higher cover percentage in the most protected area (zone A). However, its morpho-functional traits showed an inverse trend, with the largest thalli found in the moderately protected zone B and C. The remaining *Cystoseira s.l.* species were only found in zones B and C probably due to their wider area and greater habitat diversity compared to zone A. The presence of two non-indigenous species, *Asparagopsis taxiformis* and *Caulerpa cylindracea*, was observed exclusively in the less protected zones B and C. Our findings highlight the complex interactions between MPA protection levels and *Cystoseira s.l.* species conservation, with protection having, in some respects, a positive influence on selected *Cystoseira s.l.* species, indicating the importance of careful consideration in MPA design and management. Furthermore, this study provides a baseline for future monitoring of *Cystoseira s.l.* populations in light of ongoing environmental changes in the Mediterranean Sea.

## 1. Introduction

Marine Protected Areas (MPAs) have emerged as vital tools for biodiversity conservation and ecosystem management worldwide [1,2]. These protected zones serve as valuable resources for studying the effects of human activities on marine ecosystems and the potential for recovery when such pressures are reduced or eliminated [3]. Among the various indicators of ecosystem health in Mediterranean coastal waters, brown seaweeds of the genus *Cystoseira sensu lato* (*s.l.*)—including the genera *Cystoseira*, *Ericaria*, and *Gongolaria* [4,5]—have emerged as particularly significant.

In the Mediterranean rocky shores, seaweeds belonging to the genus *Cystoseira s.l.* play a valuable role as foundation species, forming complex three-dimensional habitats that support high biodiversity and provide numerous ecosystem services [6,7,8]. These seaweeds significantly enhance the structural complexity and productivity of coastal communities from the intertidal down to the upper circalittoral zone [9,10,11,12]. Additionally, they are considered useful indicators of water and ecosystem quality according to the Water Framework Directive (2000/60/EC) and the Marine Strategy Framework Directive (2008/56/EC) [13,14].

Some species of *Cystoseira s.l.* are recognized as highly impacted, particularly near urban areas, due to the interplay effects of local anthropogenic pressures (e.g., pollution, urbanization, the introduction of non-native species, overfishing, coastal aquaculture), and global climate change [7,15,16,17,18]. Consequently, the decline or loss of *Cystoseira s.l.* populations has been observed on many rocky coasts, leading to a shift from complex and productive benthic communities to less structured assemblages dominated by turf-forming algae, mussels, or sea urchin barrens [19,20], and a decrease in valuable ecosystem services [21].

Recent Mediterranean-wide studies have highlighted how multiple anthropogenic stressors affect fucoid populations across different spatial scales [22], with modeling efforts showing concerning changes in macroalgal forest distribution throughout the basin [23]. Understanding local population dynamics within protected areas is therefore crucial for developing effective conservation strategies.

While MPAs can play a critical role in the conservation of *Cystoseira s.l.* forests by providing protection from various anthropogenic impacts [24], their effectiveness in preserving these foundation seaweeds remains understudied. The protective environment within MPAs may facilitate higher reproduction rates of *Cystoseira s.l.* populations by reducing local anthropogenic stressors, facilitating conditions to successful reproduction and the establishment of new individuals [24].

We investigated the diversity and distribution of *Cystoseira s.l.* populations on the intertidal rocky shore within the “Capo Gallo-Isola delle Femmine” MPA in the central Mediterranean Sea and tested whether the diverse protection zones within the MPA may affect their conservation differently. Different *Cystoseira s.l.* species can be found in the intertidal zone and show distinct ecological preferences and distributional patterns in the Mediterranean Sea. Among them, *Ericaria amentacea* (C.Agardh) Molinari & Guiry 2020 is a photophilic species typically found in the upper sublittoral zone of exposed rocky shores, where it forms dense belts that can withstand strong wave action and high light intensities. *Cystoseira compressa* (Esper) Gerloff & Nizamuddin 1975 has a wider ecological tolerance, occurring in sheltered and moderately exposed sites in the upper sublittoral zone, often in association with vermetid platforms and tide pools. It is one of the most widespread *Cystoseira s.l.* species across the Italian coasts and throughout the Mediterranean basin [18]. *Cystoseira foeniculacea* (Linnaeus) Greville 1830 primarily inhabits the lower intertidal and upper subtidal zones, showing preference for moderately exposed areas and often found in tide pools and shallow depressions. *Ericaria brachycarpa* (J.Agardh) Molinari & Guiry 2020 and *E. crinita* (Duby) Molinari & Guiry 2020 typically occur in the upper sublittoral zone, with *E. brachycarpa* showing preference for relatively sheltered locations and tide pools, while *E. crinita* tends to occupy more exposed positions. These species are particularly sensitive to environmental changes and anthropogenic pressures, making them valuable indicators of ecosystem health. Their distribution across the Mediterranean Sea has historically been widespread, but recent decades have seen significant declines in many areas, particularly near urban centers and industrialized coastlines.

**Figure 1:**
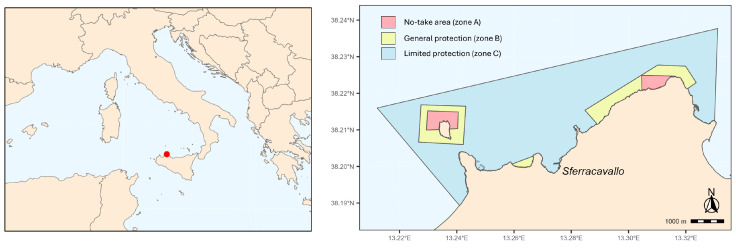
The Marine Protected Area (MPA) of “Capo Gallo—Isola delle Femmine” showing the three levels of protection. Left panel shows the MPA location (red dot) in Sicily, Italy, Mediterranean Sea. Sampling was conducted continuously along the entire coastline within the MPA boundaries, encompassing all protection zones, rather than at discrete stations, ensuring comprehensive coverage for comparative analysis of the different management regimes.

For *Ericaria amentacea*, the most abundant species and the only one present across all protection zones, we explored changes in key morpho-functional traits across the protection zones. Additionally, we documented the presence of non-indigenous seaweeds in the investigated area, providing crucial information for understanding current challenges to conservation efforts. With this study, we provide a baseline understanding of *Cystoseira s.l.* population dynamics within the “Capo Gallo-Isola delle Femmine” MPA (Figure 1) by updating a previous study [25]. These data will be essential for the MPA to plan future monitoring activities, which are necessary for effective conservation strategies in the face of global change.

## 2. Results

**Figure 2:**
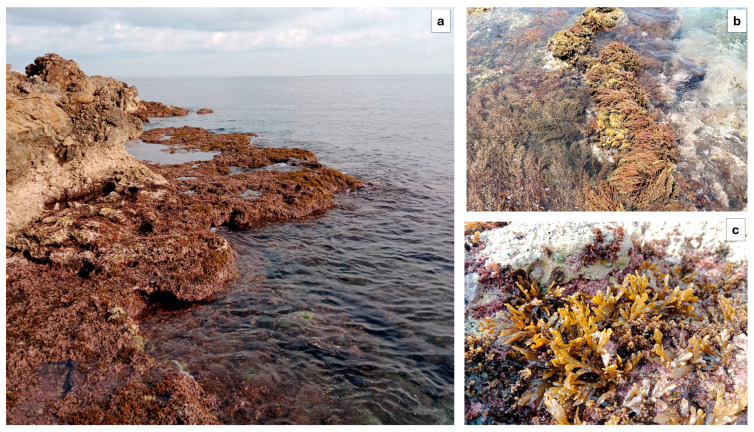
Intertidal zone within the MPA of Capo Gallo—Isola delle Femmine during low tide (**a**), with close-up views showing the *Ericaria amentacea* fringe (**b**) and *Cystoseira compressa* (**c**).

**Figure 3:**
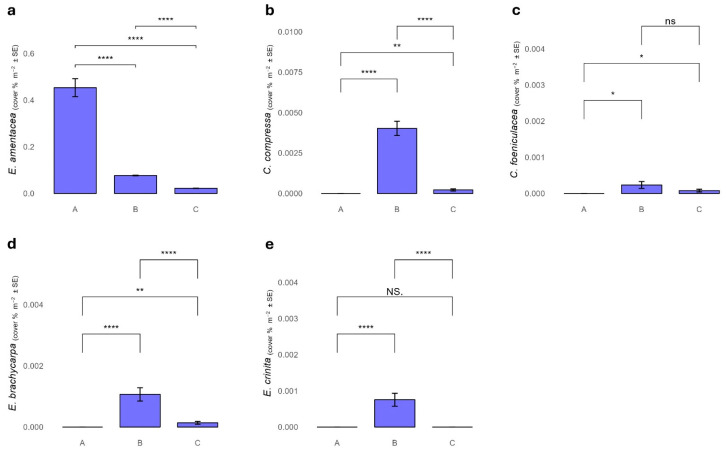
Cover percentage of *Ericaria amentacea* (**a**), *Cystoseira compressa* (**b**), *Cystoseira foeniculacea* (**c**), *Ericaria brachycarpa* (**d**), and *Ericaria crinita* (**e**) among the MPA protection zones. *p* < 0.001 ****, *p* < 0.01 **, *p* < 0.05 *, *p* ≥ 0.05 ns.

A total of five *Cystoseira s.l.* species (*Cystoseira compressa, Cystoseira foeniculacea, E. amentacea, E. brachycarpa* and *E. crinita*) were identified along the intertidal rocky shore of the MPA of Capo Gallo—Isola delle Femmine (Figure 2 and Figure 3).

*Ericaria amentacea* was mainly found on the outer edge of the vermetid reef. It was the only species among those discovered in this study that was present in all the MPA protection zones. In particular, the cover percentage of *E. amentacea* was significantly higher in zone A, and decreased in the other protection zones, with the lowest values in zone C (Figure 3a, Tables S1 and S2).

*Cystoseira compressa* was mainly discovered within intertidal pools along the vermetid reef, or close to the external edge of the vermetid reef, where substitutes *E. amentacea*. It was rarely associated with *E. amentacea*. *Cystoseira compressa* was found in B and in C zones with average cover of 2.6% and 0.44%, respectively (Figure 3b).

*Cystoseira foeniculacea*, *E. crinita* and *E. brachycarpa* were mostly found in the cuvettes of the vermetid platform. The three species, while absent in zone A, were present in lower percentages in zones B and C. Specifically, *C. foeniculacea* was found in zone B with an average percentage of 0.25%, and in zone C with a percentage of 0.18%. Ericaria brachycarpa was present in zone B with an average percentage of 0.70%, and in zone C with an average coverage of 2%, while *E. crinita* was found only in zone B with an average percentage of 0.48% (Figure 3c–e).

### 2.1. Morpho-Functional Parameters of Ericaria amentacea

**Figure 4:**
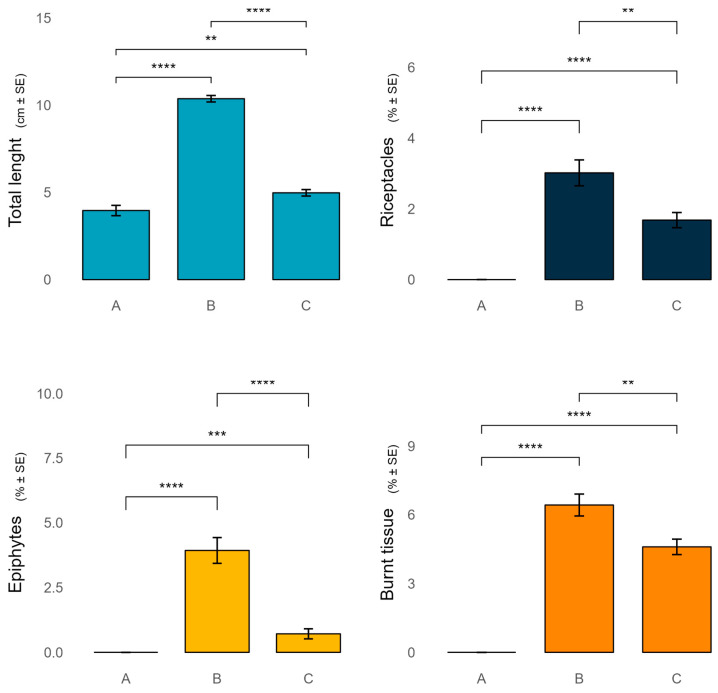
Variation in the morpho-functional parameters of Ericaria amentacea among the MPA protection zones. *p* < 0.0001 ****, *p* < 0.001 ***, *p* < 0.01 **.

Ericaria amentacea presented bigger thalli in zone B, with a total length of about 10.4 ± 0.2 cm, compared to zones C and A, where thalli were 5 ± 0.2 cm, and 4 ± 0.3 cm, length, respectively (Figure 4). The percentage of receptacles, epiphytes and burnt tissue were, in general, more present in zone B (3 ± 0.4, 4 ± 0.5, 6.4 ± 0.5%, respectively) than in zone C (1.7 ± 0.2, 0.7 ± 0.2, 4.6 ± 0.3%, respectively), and absent in zone A (Figure 4).

### 2.2. Non-Native Species

**Figure 5:**
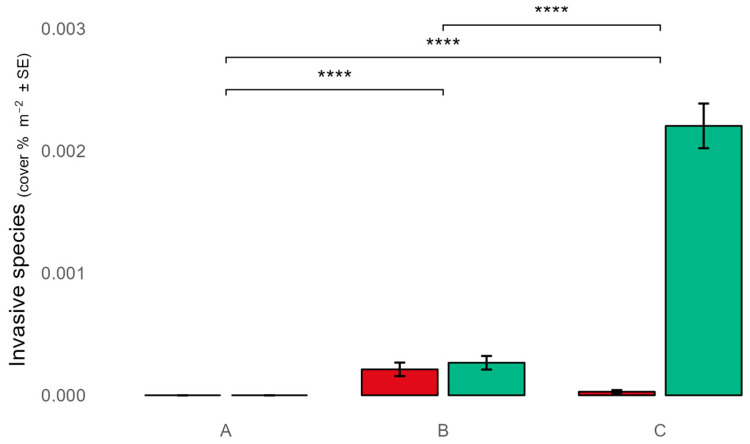
Cover percentage of *Asparagopsis taxiformis* (red), and *Caulerpa cylindracea* (green), among the MPA protection zones. *p* < 0.0001 ****.

Two species, Asparagopsis taxiformis (Delile) Trevisan 1845 and Caulerpa cylindracea Sonder 1845, were discovered, with the latter one being more abundant (Figure 5). These species were found only in zones B and C, with *C. cylindracea* being more abundant in zone C (Figure 5). Asparagopsis taxiformis was found in intertidal pools or in the outer part of the external edge of the vermetid reef, while *C. cylindracea* was mainly found in the cuvettes of the vermetid platform with thalli not longer than 1–2 cm.

## 3. Discussion

Our findings reveal a diversified pattern of *Cystoseira s.l.* species distribution across different protection zones within the MPA of “Capo Gallo—Isola delle Femmine”, highlighting the potential effectiveness of MPAs in conserving these important foundation species. We identified five *Cystoseira s.l.* species (*C. compressa*, *C. foeniculacea*, *E. amentacea, E. brachycarpa* and *E. crinita*) on the intertidal rocky shores of the MPA. This diversity underscores the ecological importance of the MPA and aligns with previous studies documenting the richness of *Cystoseira s.l.* species in Mediterranean coastal ecosystems [24,25,26]. Compared to previous research carried out into the MPA, we observed changes in *Cystoseira s.l.* composition rather than in number of species. In fact, a previous study highlighted the presence of *Cystoseira humilis* Schousboe ex Kützing 1860 [25]. We believe the absence of this species in our study may be due to its restricted range in the intertidal zone, as it is usually confined to intertidal rocky pools. It is possible that during our survey, *C. humilis* may have been retracted or less visible due to the high temperatures that intertidal pools can reach during the sampling season. However, confirming this hypothesis requires further investigation. The discrepancy in our findings underscores the importance of considering temporal factors in surveys, particularly for intertidal species that are highly subjected to variation in environmental variables. While our sampling was conducted during the period of maximum development of these species (May–June 2022), we acknowledge that extended temporal sampling throughout different seasons would provide a more comprehensive understanding of population dynamics.

We found that the distribution of these species varied significantly across the MPA’s protection zones, suggesting that the level of protection influences their abundance and composition. *Ericaria amentacea* was the most widely distributed and abundant species and was the only species present in all protection zones, aligning with previous observation [25]. However, compared to previous research, *E. amentacea* presented a significantly higher cover percentage in zone A (no-take/no-access), suggesting an improvement in its status and supporting the hypothesis that stricter protection measures can benefit *Cystoseira s.l.* populations, possibly by reducing direct human stressors and maintaining favorable environmental conditions [24]. While our results show some positive effects of protection, particularly for *E. amentacea* coverage in zone A, recent studies suggest that complete ecosystem restoration may require longer timeframes than previously anticipated [27]. The observed differences in species distribution across protection zones likely reflect the complex interaction between protection levels and local environmental conditions, as demonstrated in recent Mediterranean-wide assessments [22].

The variable abundances of *Cystoseira s.l.* species across protection zones demonstrate the complex relationship between conservation measures and species-specific ecological needs. *Ericaria amentacea* thrived in the highly protected zone A, whereas *C. compressa*, *C. foeniculacea*, *E. brachycarpa*, and *E. crinita* were more abundant in the less strictly protected Zones B and C. The observed distribution patterns of *Cystoseira s.l*. species across protected zones are most likely the result of a complex interaction of factors such as species-specific environmental tolerances, anthropogenic stressors, and interspecific competition. The geomorphological properties of the seabed have an important influence in shaping coastal marine ecosystems and, by extension, seaweed distribution. In the intertidal zone, substrate composition, topographical features, and hydrodynamic conditions all have a significant influence on the formation and composition of macroalgal communities [28]. Substrate qualities have a direct impact on algal attachment and growth, with various species preferring specific substrate types [29,30].

Furthermore, hydrodynamic conditions influenced by wave exposure and water flow patterns promote the dispersion of reproductive propagules and nutrients, influencing the distribution and diversity of macroalgal assemblages [31]. While we did not specifically include geomorphological data in our analysis, we recognize its potential importance in understanding the observed differences in macroalgal distribution among protected zones. Future research incorporating environmental monitoring (e.g., temperature, light, wave exposure) and extensive seabed geomorphology analysis will better clarify the complex interactions between abiotic factors and *Cystoseira s.l.* distribution patterns across protection zones. This multi-parameter approach would provide valuable insights for conservation and management strategies. The absence of some species (*C. foeniculacea*, *E. brachycarpa* and *E. crinita*) from zone A may be attributed to greater habitat variability (e.g., the presence of intertidal pools) and specific habitat requirements that are not met in the most strictly protected areas, rather than the level of protection.

The analysis of *E. amentacea*’s morpho-functional traits across protection zones reveals interesting patterns. Surprisingly, individuals in zone B exhibited the largest thalli, contrary to our expectation that the most protected area (zone A) would host the most well-developed specimens. This finding suggests that, while the higher cover percentage of *E. amentacea* in zone A may be facilitated by reducing human stressors such as trampling (especially during summer), which has been shown to have a negative impact on its growth [32,33], the higher area covered by moderate levels of protection, combined with greater variation in geomorphological characteristics, may provide an optimal balance of environmental conditions for *E. amentacea* growth. The higher percentages of receptacles, epiphytes, and burnt tissue in zones B and C, compared to their absence in zone A, may also indicate, in this case, that greater variation in geomorphological characteristics of these less protected areas may experience greater environmental stress or disturbance. These factors could influence the reproductive strategies and overall health of *E. amentacea* populations. The presence of burnt tissue, in particular, may be an indicator of exposure to extreme temperatures or desiccation, which could be more pronounced in areas with higher human activity [18].

The detection of two non-native species, *Asparagopsis taxiformis* and *Caulerpa cylindracea*, exclusively in zones B and C may be concerning. However, their absence from zone A suggests that strict protection measures may help resist biological invasions, possibly by maintaining more intact and resilient native communities [34]. The higher abundance of *C. cylindracea* in zone C indicates that areas with lower protection levels may be more susceptible to invasion, potentially due to increased disturbance or altered competitive dynamics.

Our findings have important implications for the management of MPAs and the conservation of *Cystoseira s.l.* species. The effectiveness of strict protection (zone A) in supporting high cover of *E. amentacea* underscores the value of no-take/no-access areas in preserving key foundation species, especially for trampling-related stressors. The variability in species responses to protection levels highlights the need for a diverse approach to MPA zoning to accommodate the requirements of different *Cystoseira s.l*. species, especially in terms of habitat suitability (e.g., intertidal pools). The presence of invasive species emphasizes the importance of monitoring and management strategies to prevent their spread.

Future restoration efforts in the MPA should consider site-specific environmental conditions and donor population characteristics [35]. Our findings at the local scale contribute to a growing body of evidence suggesting the need for both local and regional approaches to macroalgal forest conservation in the Mediterranean [23].

This study provides a baseline for future monitoring of *Cystoseira s.l.* intertidal populations in the “Capo Gallo-Isola delle Femmine” MPA. Long-term studies are needed to track changes in species distribution, abundance, and traits over time, particularly in the context of global climate change. Additionally, investigating the specific mechanisms driving the observed patterns, such as water quality, herbivory pressure, or competitive interactions, would provide valuable insights for conservation strategies.

## 4. Conclusions

In conclusion, this study provides valuable insights into the diversity and distribution patterns of *Cystoseira s.l.* species across protection zones in the “Capo Gallo-Isola delle Femmine” MPA. The findings highlight the potential effectiveness of MPAs in conserving these important foundation species, with strict protection supporting higher cover of key species like *Ericaria amentacea*. However, the findings underscore the importance of considering temporal factors in future studies, as well as incorporating environmental and geomorphological data and investigating specific mechanisms driving the observed patterns. The presence of invasive species in less protected zones underscores the importance of monitoring and management strategies. Overall, this study provides a valuable baseline for future long-term monitoring in the MPA, particularly in the context of climate change, and identifies key areas for future investigation to inform conservation strategies for these ecologically important macroalgal communities.

## 5. Material and Methods

### 5.1. Study Area

Sampling was preformed on the intertidal rocky shore within the “Capo Gallo-Isola delle Femmine” MPA (Lat: 38.213961, Long: 13.277121) located in the northwestern coast of Sicily, Italy (Figure 1). The “Capo Gallo-Isola delle Femmine” MPA, established in 2002 by the Italian Ministry of Environment and Protection of Land and Sea, affects the sea stretch between the towns of Palermo and Isola delle Femmine. Covering approximately 22 km^2^ of sea area and a coastline of about 16 km, it is bounded to the east by the gulf of Mondello and to the west by the bay of Carini. An imposing calcareous dolomitic mountain crest (Capo Gallo, 562 m a.s.l.) defines the coastal strip, resulting in a steep and rocky coastal morphology. Due to the limestone nature, flowing waters generate karst phenomena, leading to caves of high naturalistic value (Grotta dell’Olio and Grotta della Mazzara). Only towards the western part, the rocky coast assumes a flat conformation, enlivened by the presence, about 300 m from the mainland, of the Isola delle Femmine (also known as Isola di Fuori), an isolated vestige of the aforementioned calcareous ridge.

The MPA is divided into three main zones (A, B and C), each with its own level of environmental protection (Figure 1). There are two no-take/no-access zones (zone A, total area of 1 km^2^, Figure 1), one in the north sector of Isola delle Femmine and the other in the stretch of sea at the west of Capo Gallo promontory, between the Puntazza and the Capo Gallo lighthouse. Zones B and C are buffer zones where human use restrictions, including fishing, become progressively lower. In particular, there are three general protection zones (zone B, total area of 2 km^2^, Figure 1), while the remaining sea within the MPA’s border includes a partial protection area (zone C, total area of 19 km^2^, Figure 1). The MPA is also identified as Site of Community Importance (SCI, ITA020047—Fondali di Isola delle Femmine Capo Gallo).

### 5.2. Sampling

The MPA coastline was divided into sectors of 300 m each, totaling 36 stations. Each station of 300 m was divided into 12 sectors of 25 m where sampling was performed visually on 6 random replicates using a 20 × 20 cm quadrat, the minimum area recommended for sampling Mediterranean assemblages in the upper infralittoral zone [36]. In each quadrat, the percentage coverage of *Cystoseira s.l.* species was estimated using the method proposed by Dethier et al. (1993) [37], using a frame divided into 25 equal squares: we attributed a cover score from 0 to 4 to each square and then summed up scores where the taxon was present. Organisms filling < 1/4 square were given the value of 0.5. For *E. amentacea*, a series of morpho-functional characteristics (length of the cauloid, branch length, total length of the thallus, presence/absence and percentage of receptacles, and percentage of burned tissue) were also analyzed. All surveys were carried out between May and June 2022, when the thalli of *Cystoseira s.l.* species in this area reach their maximum development.

### 5.3. Data Analysis

Differences in *Cystoseira s.l.* species cover percentage among the MPA protection zones (fixed factor with 3 levels: zone A, zone B, and zone C) were tested using one-way Analysis of Variance (ANOVA). Assumptions of normality and homogeneity of variances were tested using Shapiro–Wilk and Levene’s tests, respectively. Regarding the morpho-functional parameters of *E. amentacea*, a Draftsman Plot was first created to check for high correlations among the morpho-functional parameters. Total thallus length, cauloid length, and branch length were found to be highly correlated (cor. > 0.90), as were the presence and percentage of receptacles (cor. = 0.95) (Figure S1, Table S1). Consequently, the following parameters were selected for investigating variations among the MPA’s zones: total thallus length, percentage of receptacles, burnt tissue, and presence of epiphytes.

## Data Availability

Data are contained within the article and Supplementary Materials.

## Supplementary Materials

The following supporting information can be downloaded at: https://www.mdpi.com/article/10.3390/plants13243562/s1, Table S1: ANOVA Results testing differences of *E. amentacea* cover percentage among MPA zones. Data were square root transformed to meet homogeneity of variance. *** = *p* < 0.001. Table S2: Tukey Post-hoc Test showing differences of *E. amentacea* cover percentage among MPA zones (A = zone A, B= zone B, C = zone C). *** = *p* < 0.001; Table S3: Tukey Post-hoc Test showing differences of *C. compressa* cover percentage among MPA zones (A = zone A, B= zone B, C = zone C). *** = *p* < 0.001; Table S5: ANOVA Results testing differences of *C. foeniculacea* cover percentage among MPA zones. Data were square root transformed to meet homogeneity of variance. ns = not significant.
