# Supplementary material for: Diversity and Distribution of Intertidal Cystoseira sensu lato Species Across Protection Zones in a Mediterranean Marine Protected Area
Source: Plants (Basel). 2024 Dec 20;13(24):3562. doi: 10.3390/plants13243562 (PMC11679029; doi:10.3390/plants13243562)
Supplement: Supplementary file 1 [file plants-13-03562-s001.zip › plants-3339779-supplementary.pdf]

Diversity and distribution of intertidal *Cystoseira sensu lato* species across protection zones in a Mediterranean Marine Protected Area

Francesco Paolo Mancuso [1,2]*, Gianluca Sarà [1,2] and Anna Maria Mannino [2,3]

[1] Department of Earth and Marine Sciences (DiSTeM), University of Palermo, viale delle Scienze Ed. 16, 90128 Palermo, Italy

[2] NBFC, National Biodiversity Future Center, Palermo 90133, Italy

[3] Department of Biological, Chemical and Pharmaceutical Sciences and Technologies, University of Palermo, 90123 Palermo, Italy

* Correspondence: francesco.mancuso@unipa.it

Supplementary information

Table S1- ANOVA Results testing differences of *E. amentacea* cover percentage among MPA zones. Data were square root transformed to meet homogeneity of variance. *** = p < 0.001.

	**Df**	**Sum Sq**	**Mean Sq**	**F value**	**Pr(>F)**	**Significance**
MPA.zone	2	22.524	11.262	1003.298	0	***
Residuals	2181	24.481	0.011			

Table S2- Tukey Post-hoc Test showing differences of *E. amentacea* cover percentage among MPA zones (A = zone A, B= zone B, C = zone C). *** = p < 0.001.

	**Estimate**	**Std.Error**	**t**	**p**	**Significance**
B - A	-0.34	0.01	-23.10	0	***
C - A	-0.50	0.01	-33.93	0	***

	Estimate	Std.Error	t	p	Significance
C - B	-0.16	0.00	-33.35	0	***

Table S3- ANOVA Results testing differences of *C. compressa* cover percentage among MPA zones. Data were square root transformed to meet homogeneity of variance. *** = p < 0.001.

	Df	Sum Sq	Mean Sq	F value	Pr(>F)	Significance
MPA.zone	2	0.180	0.090	58.493	0	***
Residuals	2205	3.395	0.002			

Table S4- Tukey Post-hoc Test showing differences of *C. compressa* cover percentage among MPA zones (A = zone A, B= zone B, C = zone C). *** = p < 0.001.

	Estimate	Std.Error	t	p	Significance
B - A	0.02	0.01	3.61	0.00	***
C - A	0.00	0.01	0.26	0.96	ns
C - B	-0.02	0.00	-10.69	0.00	***

Table S5- ANOVA Results testing differences of *C. foeniculacea* cover percentage among MPA zones. Data were square root transformed to meet homogeneity of variance. ns = not significant

	Df	Sum Sq	Mean Sq	F value	Pr(>F)	Significance
MPA.zone	2	0.000	0	1.35	0.259	ns

	Df	Sum Sq	Mean Sq	F value	Pr(>F)	Significance
Residuals	2205	0.302	0	NA	NA	NA
